# Population Pharmacokinetic-Pharmacogenetic (PopPK-PGx) Model of Efavirenz in HIV-1-Infected Patients

**DOI:** 10.7759/cureus.88533

**Published:** 2025-07-22

**Authors:** Katarina Vucicevic, Danica Michalickova, Bozana Obradovic, Jovan Ranin, Djordje Jevtovic, Relja Lukic, Andrew Owen, Gordana Dragovic

**Affiliations:** 1 Department of Pharmacokinetics and Clinical Pharmacy, Faculty of Pharmacy, University of Belgrade, Belgrade, SRB; 2 Department of Pharmacology, First Faculty of Medicine, Charles University and General University Hospital, Prague, CZE; 3 Department of Pharmacology, Clinical Pharmacology and Toxicology, Faculty of Medicine, University of Belgrade, Belgrade, SRB; 4 Department of Infectious Diseases, Faculty of Medicine, Clinical Centre of Serbia, Clinic of Infectious and Tropical Diseases, University of Belgrade, Belgrade, SRB; 5 Department of Gynecology and Obstetrics, Obstetrics and Gynecology Clinic "Narodni Front, Faculty of Medicine, University of Belgrade, Belgrade, SRB; 6 Department of Pharmacology and Therapeutics, Centre of Excellence in Long-Acting Therapeutics (CELT) Institute of Systems, Molecular and Integrative Biology, University of Liverpool, Liverpool, GBR

**Keywords:** adherence, cyp2b6, efavirenz, genetics, nonmem, poppk

## Abstract

Background and objectives

Efavirenz (EFV) exhibits substantial inter-patient pharmacokinetics (PK) variability. Polymorphisms in genes involved in EFV metabolism have been associated with EFV exposure, but they have not been fully implemented to inform dosing in treatment guidelines. This work aimed to develop a population pharmacokinetic-pharmacogenetic (PopPK-PGx) model of EFV in human immunodeficiency virus type 1 (HIV-1)-positive patients, and to simultaneously explore the influence of CYP2B6* *(516G>T and c.485-18C>T) and NR1I3 polymorphisms, as well as patient characteristics on EFV PK parameters. Additionally, this study aimed to simulate the combined effects of genetic polymorphisms and nonadherence patterns on EFV plasma concentrations.

Methodology

Data from 89 patients receiving 600 mg EFV once daily were analyzed using NONMEM (v7.3) (ICON Development Solutions: Ellicott City, MD). For the structural model, considering a sparse sampling design, a one-compartment model was chosen to describe the distribution of EFV concentrations, with the oral volume of distribution (V/F) and constant rate of absorption (kA) fixed to literature-reported values. Bootstrapping and normalized prediction distribution errors (NPDE) were used for internal validation of the final model. Simulation of concentration-time profiles was conducted using the final PopPK-PGx model, following the recommended dosing regimen for typical individuals, to assess the probability of achieving target concentrations (1000-4000 ng/mL) under different PGx backgrounds and the effect of various nonadherence scenarios.

Result

Typical oral clearance (CL/F) was 13.9 L/h with 13.1% interindividual variability (IIV). CYP2B6 516G>T, and CYP2B6 c.485-18C>T were associated with EFV CL/F. On average, EFV CL/F was 36.4% lower in heterozygote patients for CYP2B6 516G>T. Moreover, on average, there is a 26.8% decrease in CL/F in patients with the TT genotype of CYP2B6 c.485-18C>T. The NPDE distribution plots confirmed that the model accurately predicted EFV concentrations. For individuals carrying either the CYP2B6 516G>T or CYP2B6 c.485-18C>T polymorphisms, missing two consecutive standard doses of 600 mg/day was estimated to be sufficient to drive EFV concentrations out of the therapeutic range, while for those not carrying any CYP2B6 polymorphisms, one missed dose was estimated to result in EFV concentrations falling below the therapeutic range.

Conclusion

These findings support the need for genotype-adherence-guided EFV dose adjustments to achieve optimal therapeutic levels. The CYP2B6 516G>T and c.485-18C>T variants were found to significantly reduce efavirenz rate of elimination, resulting in prolonged drug exposure. This insight could inform personalized strategies to maintain therapeutic concentrations in patients with varying adherence patterns.

## Introduction

Efavirenz (EFV) is a non-nucleoside reverse transcriptase inhibitor (NNRTI) widely used in combination antiretroviral therapy (cART) for the treatment of human immunodeficiency virus type 1 (HIV-1) infection [1]. According to the European AIDS Clinical Society (EACS), EFV is no longer the recommended first-line therapy but remains an alternative option for initial treatment in ART-naïve patients [2]. Moreover, the Department of Health and Human Services (DHHS) guidelines for the use of antiretroviral agents in HIV-1-infected adults and adolescents recognize EFV-based regimens as the recommended initial ART regimen in certain clinical situations [3]. Furthermore, the World Health Organization (WHO) recommends a low-dose regimen of EFV (400 mg) combined with a nucleoside reverse transcriptase inhibitor (NRTI) backbone as an alternative first-line treatment for adults and adolescents living with HIV-1 who are initiating ART [4].

Despite its proven efficacy, EFV exhibits significant interindividual variability (IIV) in pharmacokinetics (PK), which can impact therapeutic outcomes and the occurrence of adverse effects. Genetic polymorphisms in drug-metabolizing enzymes and nuclear receptors, along with patient-specific factors, have been implicated in this variability [5,6]. Nonlinear mixed effects modelling is a well-understood approach for identifying sources of variability and incorporating influential covariates in the models. Population PK (PopPK) models can be further used to reconstruct individual concentration-time profiles, as well as to simulate different dosing schemes (including nonadherence patterns) and potential clinical scenarios, thereby assisting in clinical decision-making. Cytochrome P450 2B6 (CYP2B6) is the primary enzyme responsible for EFV metabolism, and several single-nucleotide polymorphisms (SNPs) in CYP2B6have been associated with EFV plasma concentrations [5,6]. Even though polymorphisms in CYP2B6(in particular 516G>T) have been extensively investigated using the PopPK methodology, there are still some inconsistencies across studies, partly due to differences in study design, as well as due to the inclusion of diverse populations with varying allele frequencies [7-12]. Additionally, the constitutive androstane receptor (CAR, encoded by NR1I3) regulates the expression of CYP2B6, suggesting that polymorphisms in NR1I3* *may further modulate CYP2B6-mediated metabolism of EFV [13,14]. EFV itself can activate CAR, creating a feedback mechanism that influences its own clearance [15]. The clinical relevance of NR1I3variants remains less established and is likely modest compared to the strong effects of CYP2B6polymorphisms [16].

Suboptimal adherence to EFV-based therapy can result in drug concentrations falling below the therapeutic threshold. Several factors contribute to this risk, including variability in drug metabolism due to genetic differences. In such cases, intermittent dosing or missed doses can lead to insufficient viral suppression, allowing ongoing viral replication in the presence of subinhibitory drug levels. In addition, this promotes the selection of resistant viral variants. Hence, understanding the interplay between genetic variability and nonadherence patterns in influencing EFV exposure is essential to fully appreciate the clinical relevance of PGx. Identifying patients at risk through PGx profiling, combined with targeted adherence support interventions, may represent key strategies to prevent the development of drug resistance and treatment failure.

The aim of this study was to develop a PopPK-PGx model of EFV in HIV-1 positive patients, simultaneously assessing the combined influence of CYP2B6* *(516G>T and c.485-18C>T) and NR1I3 polymorphisms and patient characteristics on EFV PK parameters. Additionally, this study aimed to simulate the combined effects of nonadherence and genetic polymorphisms on EFV plasma concentrations, providing potential strategies for genotype-guided dose optimization.

## Materials and methods

PopPK-PGx model development

The current analysis utilized data from a published study [17]. Inclusion criteria for this analysis were adult patients (aged ≥18 years) of Caucasian ethnicity with confirmed HIV-1 diagnosis, receiving EFV-based ART for a minimum of three months, treated at the outpatient HIV/AIDS Center at the Clinic for Infectious and Tropical Diseases, University of Belgrade Teaching Hospital, Belgrade, Serbia. Exclusion criteria were as follows: incomplete pharmacokinetic or clinical data, concomitant use of antituberculosis medications, and other drugs known to significantly interact with EFV metabolism, individuals under 18 years of age, and pregnant women. All participants provided written informed consent prior to enrollment.

A population PK analysis was carried out using NONMEM version 7.4 (ICON Development Solutions: Ellicott City, MD) and PsN v3.4.2 (Uppsala, Sweden: Pharmacometrics Research Group, Department of Pharmaceutical Biosciences, Uppsala University), both running under Pirana v2.9.0 (Princeton, NJ: Certara USA, Inc.). Modelling was performed using the first-order conditional estimation method with interaction (FOCE-I). R v4.2.2 (Vienna, Austria: R Foundation for Statistical Computing) was used for data visualization and model diagnostics.

Model development was carried out in three steps. For the structural model, considering a sparse sampling design with one sample obtained per patient, a one-compartment model was chosen to describe the distribution of EFV concentrations incorporated in ADVAN2 TRANS2 routine. Given the constraints in the reliable estimation of multiple disposition phases that a more complex model would require, a one-compartment model was considered appropriate to capture the primary PK characteristics of EFV while avoiding model overparameterization. To stabilize the model and enable estimation of oral clearance (CL/F) as the primary parameter of interest, the volume of distribution (V/F) and absorption rate constant (k_A_) were instead fixed to literature-reported values as follows: 237 L/70 kg [9,11,18,19] and 0.3 h^-1^, respectively [20]. First-order elimination of EFV was assumed. IIV was tested for CL, assuming log-normal distribution with estimated variance. For the residual error model, proportional, additive, and combination error models were tested.

For the development of the covariate model, a stepwise covariate modelling procedure was executed. Continuous covariates (body weight, body mass index) were tested in linear and power functions, whereas categorical covariates (sex, smoking, CYP2B6* *516G>T {rs3745274}, CYP2B6* *c.485-18C>T {rs4803419}, NR1I3* *c.540C>T {rs2307424}, and NR1I3* *c.152-1089T>C {rs3003596}) were tested by estimating the parameter value for one category as a fraction of the parameter value for the other category. These were tested as covariates for CL. For model selection, a reduction in the objective function value (OFV) of more than 3.84 points between nested models (p<0.05) was considered statistically significant, assuming a chi-square test distribution. Further criteria for model selection were relative standard error (RSE) of the estimates of structural model parameters less than 30%, physiological plausibility of the parameter estimates, reduction in the IIV of CL, and absence of bias in goodness-of-fit (GOF) plots.

Finally, for internal validation of the final model, bootstrapping and normalized prediction distribution errors (NPDE) were used. To test the stability of the model, a bootstrap analysis was carried out. For this, 1000 replicates of the original data were created, and the parameter estimates for each sample were re-estimated. The median and 95% confidence interval (CI) acquired for each of the parameters estimated from the bootstrap samples were then compared with the final model parameter estimates. For NPDE, the original dataset was simulated 1000 times, after which the observed concentrations were compared with the range of simulated values using the NPDE package developed for R [21].

Model-based dose simulations

To illustrate the significance of our findings, simulations were conducted using the final PopPK-PGx model to assess the probability of achieving the target concentrations (1000-4000 ng/mL). In total, 1000 simulations for a typical individual with a body weight of 76 kg and different combinations of CYP2B6polymorphisms - 516G>T (GG vs. GT) and c.485-18C>T (CC vs. TT) - were performed for the standard dose of 600 mg daily. Moreover, simulations were also performed for a typical individual with different combinations of CYP2B6polymorphisms to determine the number of missed doses required for EFV concentrations to fall below 1000 ng/mL in the steady state.

## Results

PopPK-PGx model

In total, 89 patients were included with a single blood steady-state EFV sample obtained from 2.83 to 13.42 hours (concentration vs. time after last dose is depicted in appendix 1) following 600 mg once daily dosing. Demographic details are presented in Table 1.

**Table 1:** Clinical characteristics of the patients included in the analysis. *Values are presented as median (interquartile range), unless stated otherwise.

Parameters (units)	Values*
Body weight (kg)	76 (66-85)
Sex, male; n (%)	21 (23.60)
Age (years)	40 (32-48)
Smoking, n (%)	60 (67.41)
Carriers of CYP2B6 c.516G>T (rs3745274); n (%)	30 (33.71)
Carriers of CYP2B6 c.485-18C>T (rs4803419); n (%)	12 (13.48)
Carriers of NR1I3 c.540C>T (rs2307424); n (%)	53 (59.60)
Carriers of NR1I3 c.152-1089T>C (rs3003596); n (%)	58 (65.17)

As previously stated, observed EFV concentrations were best described by a one-compartment model with log-normally distributed IIV on CL. Typical CL/F was estimated at 13.9 L/h (4.4% RSE), while residual variability was explained by a standard deviation of 0.25 (11%). IIV in CL/F was best described by an exponential model, and the estimated value was 13.1% (35% RSE). CYP2B6516G>T and CYP2B6c.485-18C>T were associated with EFV CL/F. Our results indicate that EFV CL/F was 36.4% lower in patients with the GT (n=30) genotype compared to GG (n=60) for CYP2B6516G>T. In addition, the model suggested a 26.8% decrease in CL/F in patients with TT genotype (n=12) of CYP2B6c.485-18C>T. Smoking status was excluded from the model in the backward step of covariate model building. The estimates of the final model parameters are presented in Table 2. The estimated parameter values demonstrated satisfactory precision, with RSE values below 30%, apart from IIV for CL/F, which was 35%.

**Table 2:** Estimated parameters of the final efavirenz (EFV) model. RSA: binary parameter indicating whether the patient is a carrier of CYP2B6 516G>T (1) or not (0); RSB: binary parameter indicating whether patient is a carrier of CYP2B6 c.485-18C>T (1) or not (0); CLp: population clearance value; F: bioavailability; CV: coefficient of variation

Parameter (units)	Final model (RSE%)	Bootstrap (2.5-97.5 percentiles)
Fixed effects		
CL/F (L/h) = CLp * (1 +θ_RSA_)^RSA^ × (1 +θ_RSB_)^RSB^		-
CLp (L/h)	13.9 (4)	13.9 (12.73 to 15.21)
θ_RSA_	-0.364 (14)	-0.368 (-0.457 to -0.255)
θ_RSB_	-0.268 (16)	-
Inter-individual variability		
CV_CL_ (%)	13.1 (35)	13.4 (3.24 to 20.9)
Residual unexplained variability		
Proportional error	0.25 (11)	0.244 (0.182 to 0.299)

**Figure 1:**
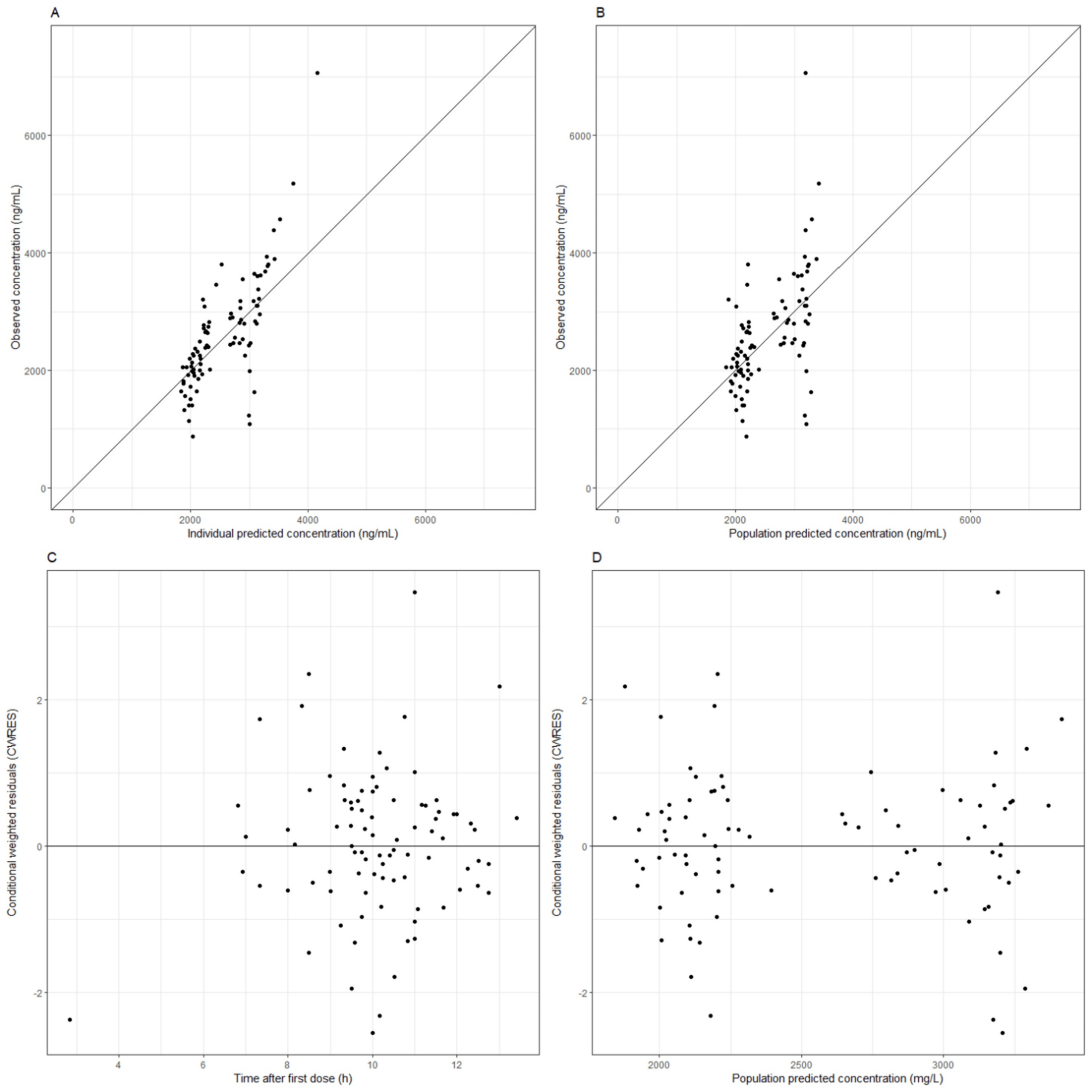
Basic goodness-of-fit (GOF) plots of the final model. (A) Population-predicted vs. observed efavirenz (EFV) concentration. (B) Individual-predicted vs. observed EFV concentration. (C) Conditional weighted residuals (CWRES) vs. time after last dose. (D) CWRES vs. population-predicted concentration.

The final model effectively described the data, with no bias in the goodness-of-fit (GOF) plots (Figures 1A-1D). Additionally, median parameter values obtained through the bootstrap procedure were within 10% of those from the final model fit, confirming the robustness of the model results. The final PopPK-PGx model was also validated by numerical and simulation-based NPDE. The NPDE distribution plots confirmed that the model accurately predicted EFV concentrations, showing no trends over time or across the observed concentration ranges (appendix 2).

Model-derived dosing implications

**Figure 2:**
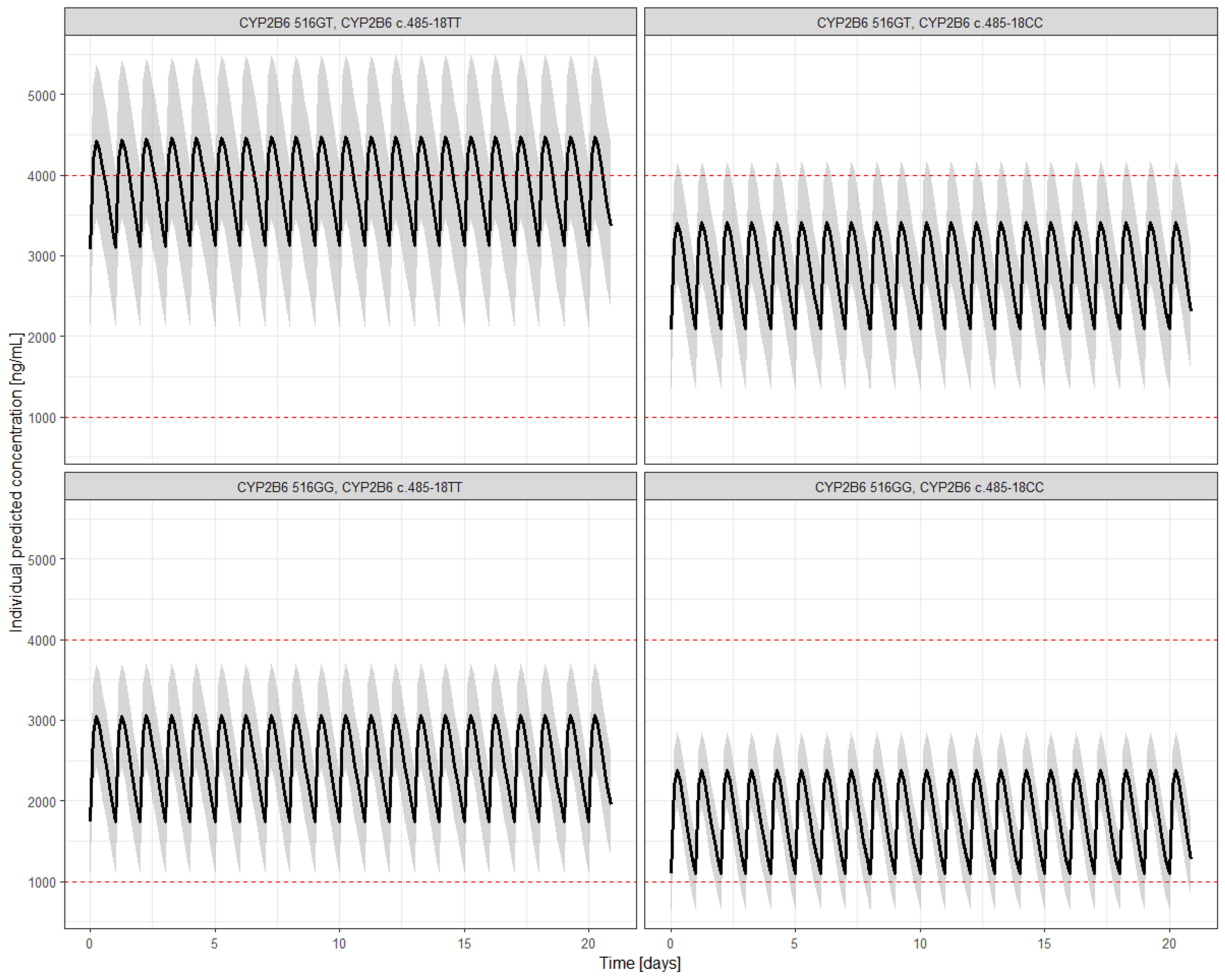
Simulations of concentration-time profile of efavirenz (EFV) for different combinations of covariates based on final model.

To illustrate the implications of our results regarding different PGx backgrounds, simulations were performed with standard EFV dosing and the final PopPK-PGx model, including IIV for CL (Table 3 and Figure 2). The results show that patients carrying both the CYP2B6516G>T and CYP2B6c.485-18C>T polymorphisms exhibit the highest EFV concentrations, with 5.3% of the patients having a trough concentration (C_trough_) exceeding the therapeutic range (1000-4000 ng/mL). Carriers of either the CYP2B6516G>T or CYP2B6c.485-18C>T polymorphism generally maintain C_trough_ within the therapeutic range. In contrast, individuals without any CYP2B6 polymorphism have the highest likelihood of subtherapeutic C_trough_ levels, with approximately 35% of the patients falling below the recommended range.

**Table 3:** Covariates influence on trough concentration (Ctrough) based on simulation using developed efavirenz (EFV) model.

Polymorphism	Median C_trough_ (95% CI)	Patients with C_trough_ <1000 (%)	Patients with C_trough_ > 4000 (%)
CYP2B6 516GT, CYP2B6 c.485-18TT	3072.75 (2208.258-4187.052)	0	5.3
CYP2B6 516GT, CYP2B6 c.485-18CC	2140.05 (1501.855-2910.855)	0	0
CYP2B6 516GG, CYP2B6c.485-18TT	1757.15 (1221.467-2500)	0.02	0
CYP2B6 516GG, CYP2B6 c.485-18CC	1179.4 (793.94-1720)	34.8	0

**Figure 3:**
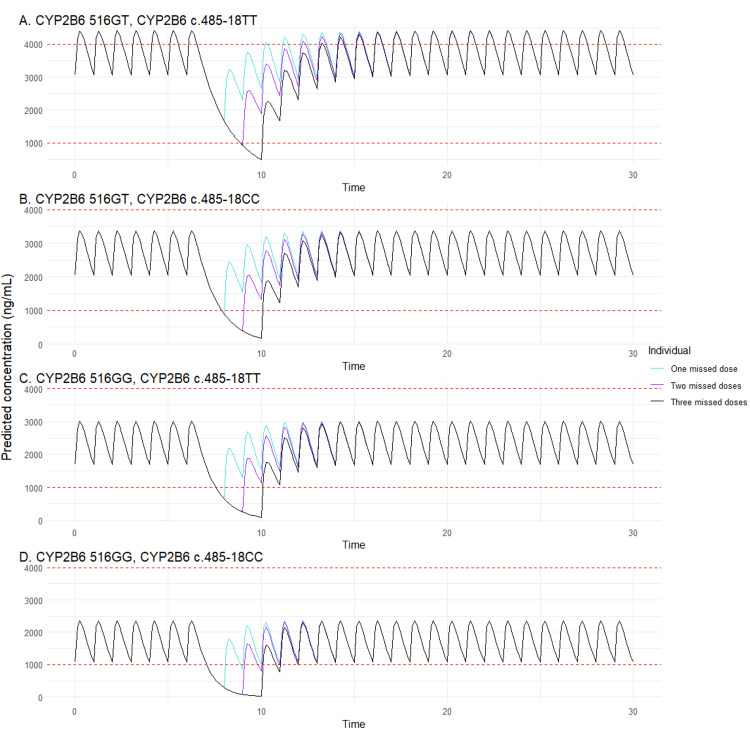
Simulations of the concentration-time profile of efavirenz (EFV) considering different nonadherence schemas after standard dosing.

We also employed the model to evaluate how adherence may affect EFV concentrations, assessing the number of missed doses required for steady-state EFV concentrations to fall below 1000 ng/mL in different scenarios. Simulations of the recommended dosing regimen for typical individuals with different PGx backgrounds and the effect of missed doses are presented in Figures 3A-3D. For an individual carrying both the CYP2B6516G>T and CYP2B6c.485-18C>T polymorphisms, missing three consecutive doses of EFV was estimated to be required for concentrations to fall out of the therapeutic range at steady state. In contrast, for individuals carrying either the CYP2B6 516G>T or CYP2B6 c.485-18C>T polymorphisms, missing two consecutive doses was estimated to be sufficient to drive EFV concentrations out of the therapeutic range. For individuals not carrying any CYP2B6 polymorphisms, one missed dose was estimated to result in EFV concentrations falling below the therapeutic range.

## Discussion

While our initial study assessed the association between genetic polymorphisms and single-timepoint EFV plasma concentrations, it did not capture the full complexity of the EFV PK profile [17]. By applying PopPK methodology, we were able to quantify IIV in EFV CL/F and systematically evaluate the combined impact of various covariates on EFV exposure. We have now developed a PopPK-PGx model of EFV in our cohort of patients with HIV-1. Our analysis showed that CYP2B6516G>T and CYP2B6c.485-18C>T polymorphisms were associated with lower EFV CL/F. Unlike static concentration measurements, PopPK analysis enables dynamic characterization of drug behavior over time, offering a more comprehensive and mechanistic understanding of how polymorphisms influence EFV disposition in real-world settings. Moreover, the presented simulations provide a rationale for integrating pharmacogenetics into clinical practice in the context of patient nonadherence patterns.

Due to the sparse study design, a one-compartment model was used to describe EFV distribution. This is in line with the previous studies [18,22]. Moreover, due to the sparse study design, we could not estimate the kA and V/F; therefore, their values were fixed to 0.3 h-1 and 237 L for a body weight (BW) of 70 kg [9,11,18-20]. Overall, CL/F estimate (13.9 L/h) in our study was consistent with estimated values from the previous studies, ranging from 4 to 15 L/h [11,18-20,22].

EFV CL/F was 36.4% lower in patients with the GT genotype compared to the GG genotype of CYP2B6* *516G>T polymorphism and 26.8% lower in patients with the TT genotype compared to the CC genotype for CYP2B6* *c.485-18C>T. The CYP2B6* *516G>T polymorphism represents a well-established loss-of-function allele [23]. It was among the first to be studied extensively and is consistently linked to increased EFV exposure, as well as reduced CL and metabolism. However, the effects of the CYP2B6c.485-18C>T polymorphism on PK of EFV are less studied. The use of a nonlinear mixed-effects modeling approach in this context appears to be limited. Bienczak et al. found no effect of CYP2B6c.485-18C>T polymorphism on PK of EFV in Ugandan and Zambian children [24], whereas Bertrand et al. found CYP2B6* *c.485-18C>T polymorphism to decrease EFV CL by 30% in adult patients EFV and antituberculosis treatment in Cambodia [18]. The latter study is consistent with our presented analysis. In addition, our findings align with Clinical Pharmacogenetics Implementation Consortium (CPIC) guidelines, which recommend genotype-guided dosing of EFV based on CYP2B6516G>T polymorphism due to its well-established impact on drug CL and the risk of central nervous system toxicity [16]. In our study, the 516G>T variant was associated with a 36.4% reduction in CL/F, supporting the clinical relevance of this polymorphism. Notably, we also identified a significant association with CYP2B6c.485-18C>T, which is not currently included in CPIC guidance. These results suggest the potential for refining genotype-based dosing algorithms to incorporate additional variants that may influence EFV PK.

The number of PopPK studies exploring the effects of NR1I3 c.540C>T and NR1I3 c.152-1089T>C on EFV pharmacokinetics appears to be limited. No impact of these polymorphisms were observed in the current analysis. There are conflicting findings regarding the impact of NR1I3c.540C>T on EFV concentrations and PK as follows: T homozygosity was linked to lower EFV concentrations in Chilean HIV-1 patients [25]; however, studies in African and Caucasian populations failed to confirm this association [17,26,27]. Subsequently, a meta-analysis of NR1I3 polymorphisms on EFV plasma concentrations suggested that individuals with the TT genotype exhibit significantly lower EFV concentrations compared to those homozygous for the C allele [5]. However, the same meta-analysis did not identify a significant association between NR1I3rs3003596 and EFV concentrations, and the CPIC guideline does not currently include NR1I3polymorphisms in dosing recommendations for EFV [16]. These findings are consistent with our results.

Model-derived simulations indicated that patients with both CYP2B6* *516G>T and CYP2B6c.485-18C>T polymorphisms have the highest EFV concentrations, with 5.3% exceeding the therapeutic range. In line with this, patients with these genotypes were estimated to require three consecutive missed doses for EFV concentrations to fall below the therapeutic range at steady state. Conversely, individuals without these genotypes were estimated to be at highest risk of subtherapeutic levels, with 35% patients falling below the recommended threshold. This implies that these patients are at the highest risk of EFV underexposure. Indeed, the simulations for a typical individual without any CYP2B6 polymorphism estimated that missing just one dose would lead to EFV concentrations falling outside the therapeutic range. Finally, carriers of either, but not both, polymorphisms were estimated to maintain concentrations within the range, with two consecutive missed doses resulting in EFV concentrations below the therapeutic range. These findings illustrate important clinical implications of lower adherence, as EFV underexposure in HIV-1 patients can detrimentally affect viral suppression and can also drive the emergence of resistance.

This study has several limitations that should be considered when interpreting the results. First, it employed a sparse sampling design with only one sample collected per patient, which limited the ability to estimate certain PK parameters, such as V/F and k_A_. These parameters were therefore fixed to values reported in the literature. Despite this, the relative standard error (RSE) values for the estimated model parameters and the diagnostic plots confirmed the model's robustness and adequate fit to the observed data. Second, the study population consisted exclusively of white Caucasian patients. While this approach minimized the risk of population stratification, it also restricts the generalizability of the findings to more diverse global populations. For example, the allele frequencies of CYP2B6516G>T and CYP2B6c.485-18C>T in our cohort were comparable to those reported in other European populations but differed significantly from frequencies observed in African and American populations [17,28]. Given that the majority of people living with HIV are located in Africa, further studies in more genetically diverse populations are warranted to validate and expand upon these findings.

## Conclusions

This population pharmacokinetic and pharmacogenetic analysis of efavirenz in HIV-1 patients identified two genetic variants, CYP2B6516G>T and CYP2B6c.485-18C>T, that significantly reduce EFV clearance by 36.4% and 26.8%, respectively. Simulations after standard dosing showed that patients homozygous for both variants maintain higher EFV concentrations and may require missing three consecutive doses before drug levels fall below the therapeutic threshold. Individuals with only one of these variants may need to miss two doses, while those without these polymorphisms reach subtherapeutic levels after just one missed dose. These findings provide important insights that could support personalized efavirenz dosing and adherence strategies in clinical practice.

Future research should focus on validating these findings in larger and ethnically diverse cohorts to improve generalizability. Additionally, incorporating longitudinal nonadherence data and investigating the long-term clinical impact of genetic variability on treatment outcomes would be valuable. Exploring other PGx markers and their interactions may further refine personalized EFV therapy. Such studies have the potential to shape more precise and effective treatment guidelines for people living with HIV-1.

## Figures and Tables

**Figure 4:**
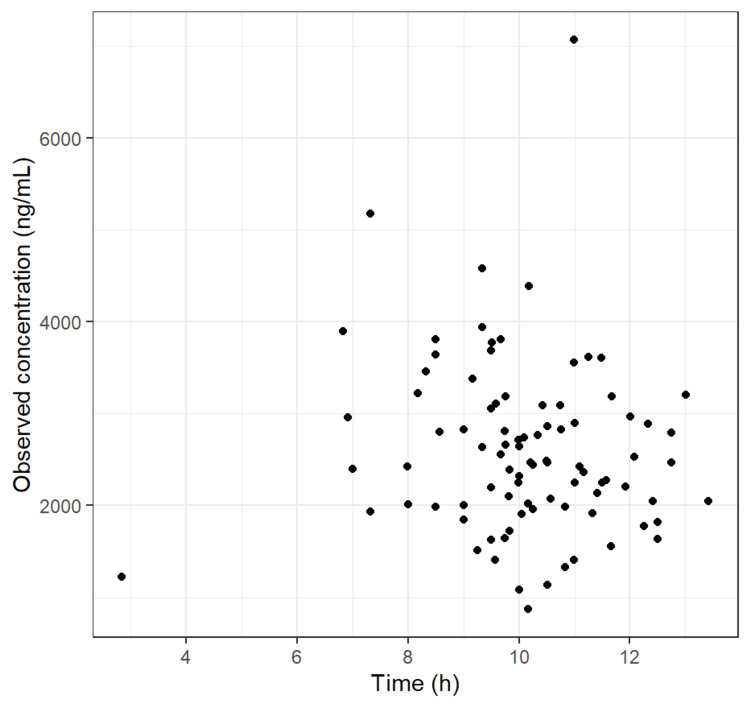
Observed concentrations of efavirenz (EFV) vs. time after dose.

**Figure 5:**
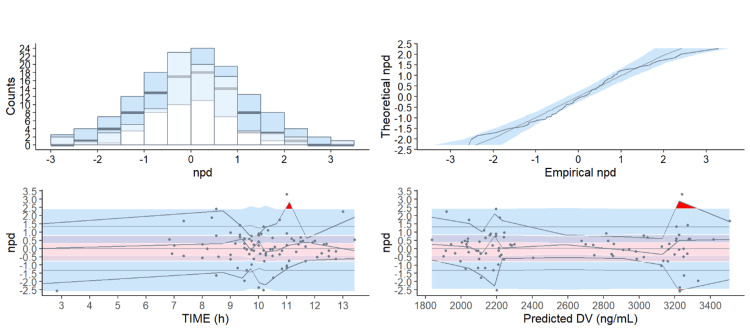
Normalized prediction distribution error (NPDE) diagnostics for the final efavirenz (EFV) model. At the top are the QQ plot and histogram of the NPDEs in the overall dataset. The red dotted line and blue shaded areas show the expected trends and 95% confidence intervals (CI) of these trends, while the dark blue lines and bars show the observed NPDE distributions. At the bottom, individual NPDE values for each observation are plotted against time and predicted concentrations, using distinct symbols. The solid lines in the bottom graphs indicate the mean (red) and the 95% percentiles (blue) of the NPDEs, and the shaded areas are the simulated 95% confidence intervals of the NPDE median (red) and 95% percentiles (blue), while the dotted red and blue lines show the expected values for the median and 95% percentiles. The distribution of NPDEs has a mean of 0.058 and a variance of 1.009, both of which are not significantly different from the expected values of 0 (p=1) and 1 (p=1), respectively.

## Appendices

Appendix 1

Appendix 2 
